# Cardiometabolic therapy for rhythm control: a case report of refractory atrial fibrillation resolving after initiation of semaglutide for obesity

**DOI:** 10.1093/ehjcr/ytag727

**Published:** 2026-09-28

**Authors:** Frederike Marie Weitkamp, Mirela Vatic, Stephan von Haehling, Constanze Schmidt, Felix Wiedmann

**Affiliations:** Department of Cardiology and Pneumology, University Medical Center Goettingen, Robert-Koch-Street 40, Goettingen D-37075, Germany; Department of Cardiology and Pneumology, University Medical Center Goettingen, Robert-Koch-Street 40, Goettingen D-37075, Germany; DZHK (German Center for Cardiovascular Research), Partner Site Lower Saxony, University of Goettingen, Rober-Koch-Street 42a, Goettingen D-37075, Germany; Department of Cardiology and Pneumology, University Medical Center Goettingen, Robert-Koch-Street 40, Goettingen D-37075, Germany; DZHK (German Center for Cardiovascular Research), Partner Site Lower Saxony, University of Goettingen, Rober-Koch-Street 42a, Goettingen D-37075, Germany; Department of Cardiology and Pneumology, University Medical Center Goettingen, Robert-Koch-Street 40, Goettingen D-37075, Germany; DZHK (German Center for Cardiovascular Research), Partner Site Lower Saxony, University of Goettingen, Rober-Koch-Street 42a, Goettingen D-37075, Germany; Department of Cardiology and Pneumology, University Medical Center Goettingen, Robert-Koch-Street 40, Goettingen D-37075, Germany; DZHK (German Center for Cardiovascular Research), Partner Site Lower Saxony, University of Goettingen, Rober-Koch-Street 42a, Goettingen D-37075, Germany

**Keywords:** Atrial fibrillation, Cardiometabolic therapy, Case report, Obesity, Semaglutide

## Abstract

**Background:**

Obesity is a major modifiable risk factor for atrial fibrillation (AF) and has been linked to processes involved in atrial structural and electrical remodelling. Glucagon-like peptide-1 (GLP-1) receptor agonists reduce cardiovascular events in patients with type 2 diabetes at high cardiovascular risk and are increasingly regarded as cardiometabolic therapies. Whether these agents modify the trajectory of atrial cardiomyopathy or exert direct antiarrhythmic effects beyond their metabolic actions remains an area of active investigation.

**Case summary:**

A 50-year-old man with obesity (body mass index 34.9 kg/m^2^) and persistent AF presented with persistent symptomatic episodes despite four prior pulmonary vein isolation procedures and repeated electrical cardioversions. Rhythm monitoring was performed continuously using a wearable device since 2023, documenting recurrent episodes lasting 8–30 h with a modified European Heart Rhythm Association (mEHRA) class IIb–III symptom burden. Echocardiography demonstrated preserved left ventricular systolic function and left atrial enlargement. In January 2026, once-weekly subcutaneous semaglutide was initiated for weight management (0.25 mg/week with subsequent dose escalation). While a weight reduction of 14 kg was observed within 6 months, AF episodes and symptoms ceased immediately after treatment initiation and have not recurred during follow-up.

**Conclusion:**

This case describes cessation of recurrent AF episodes following initiation of semaglutide in a patient with obesity and prior ablation failure. Potential mechanisms may include cardiometabolic effects as well as direct myocardial effects on sodium and calcium handling that have been described experimentally. Although a causal relationship cannot be established from a single observation, this finding may serve as a hypothesis-generating signal and provide a rationale for prospective studies evaluating cardiometabolic therapies as potential adjuncts in rhythm control strategies.

Learning points

- Obesity is associated with AF and represents a modifiable therapeutic target
- The European Society of Cardiology guidelines emphasize weight reduction as a critical intervention to reduce AF burden
- GLP-1 receptor agonists reduce cardiovascular events in patients with type 2 diabetes at high cardiovascular risk and are increasingly regarded as cardiometabolic therapies
- GLP-1 receptor agonists may represent a promising adjunctive strategy in obesity-associated AF through weight reduction and broader cardiometabolic effects. However, their role in rhythm control requires confirmation in prospective studies

## Introduction

Atrial fibrillation (AF) is the most common sustained cardiac arrhythmia, with obesity representing an important and potentially modifiable risk factor.^1^ Excess adiposity promotes atrial cardiomyopathy through structural remodelling, inflammation, and expansion of epicardial adipose tissue, thereby facilitating arrhythmogenesis.^2,3^ Weight reduction has been shown to decrease AF burden and improve rhythm control outcomes, including after catheter ablation.^4,5^

Glucagon-like peptide-1 (GLP-1) receptor agonists are established cardiometabolic therapies for obesity and type 2 diabetes with proven cardiovascular benefits.^6,7^ However, their potential impact on cardiac rhythm control remains insufficiently characterized.

Here, we report cessation of recurrent AF following initiation of semaglutide therapy for weight management in a patient with prior ablation failure (Summary Fig.).

## Summary figure

Reduction of atrial fibrillation burden upon initiation of semaglutide

eCV, electrical cardioversion; PVI, pulmonary vein isolation; RF, radiofrequency ablation.

Alt text, summary figure: Timeline (2013–2026) showing recurrent smartwatch-documented atrial fibrillation from August 2023 to December 2025 despite repeated pulmonary vein isolation procedures in 2013, 2023, and 2024 and electrical cardioversions, followed by initiation and dose escalation of semaglutide in January 2026 and immediate cessation of further documented atrial fibrillation episodes.

**Figure:**
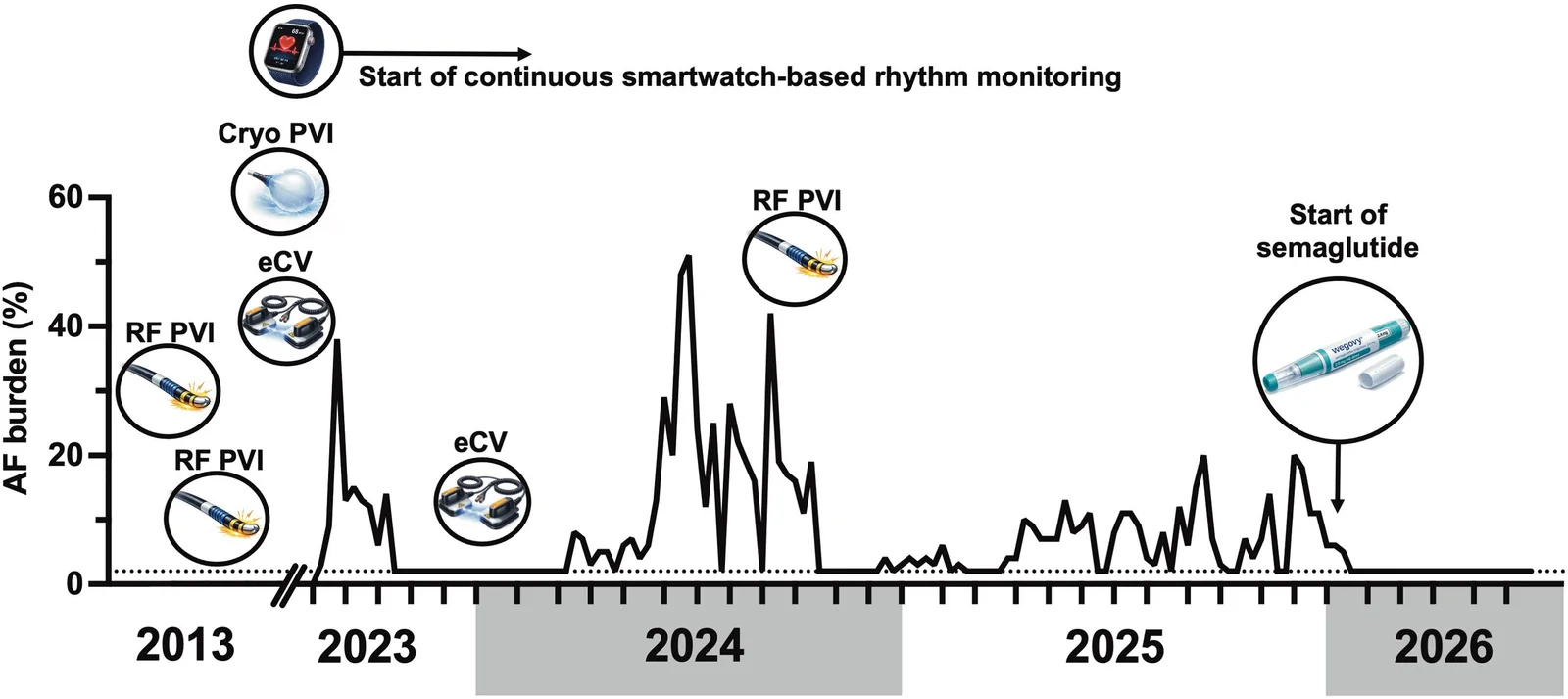


## Case presentation

A 50-year-old patient with obesity (body mass index 34.9 kg/m^2^) and a history of persistent AF presented with recurrent symptomatic arrhythmic episodes despite multiple prior rhythm control interventions (see Timeline). During episodes of AF, the patient reported dizziness, dyspnoea, and palpitations, severe enough to interrupt daily activities. Relevant cardiovascular risk factors included conservatively managed arterial hypertension (aHT), dyslipidaemia, and a history of nicotine abuse (7.5 pack-years, cessation in 2007). Diabetes mellitus was excluded (HbA1c 5.6%). Screening for obstructive sleep apnoea was recommended but has not yet been performed. Apart from obesity, physical examination revealed no clinically significant abnormalities.

Atrial fibrillation was first diagnosed in 2012. Two radiofrequency pulmonary vein isolation procedures were performed in 2013. Recurrence in 2023 led to a second redo pulmonary vein isolation (cryoballoon ablation). Two electrical cardioversions were required during follow-up. In October 2024, the patient was referred for a fourth ablation procedure.

Continuous smartwatch documentation demonstrated AF episodes during symptoms (*Figure 1A*). Resting electrocardiography showed sinus rhythm at 75 b.p.m. (*Figure 1B*). The resting electrocardiogram demonstrated a first-degree atrioventricular block, right bundle branch block, and left posterior fascicular block (*Figure 1B* and Supplementary material online, *Figure S1*). Echocardiography performed during sinus rhythm demonstrated preserved left ventricular systolic function (60%) with mild septal hypertrophy, normal diastolic indices, mild mitral valve regurgitation with moderate left atrial dilatation (45 mm; *Figure 1C–E*), and normal right ventricular function (tricuspid annular plane systolic excursion (TAPSE) 19 mm; Supplementary material online, *Table S1*). Electroanatomical mapping demonstrated reconnection of the right superior pulmonary vein (RSPV) whereas the remaining atrial substrate appeared electrically preserved (*Figure 1F* and *G*). Consecutively re-isolation of the RSPV was achieved (*Figure 1H*).

**Figure 1:**
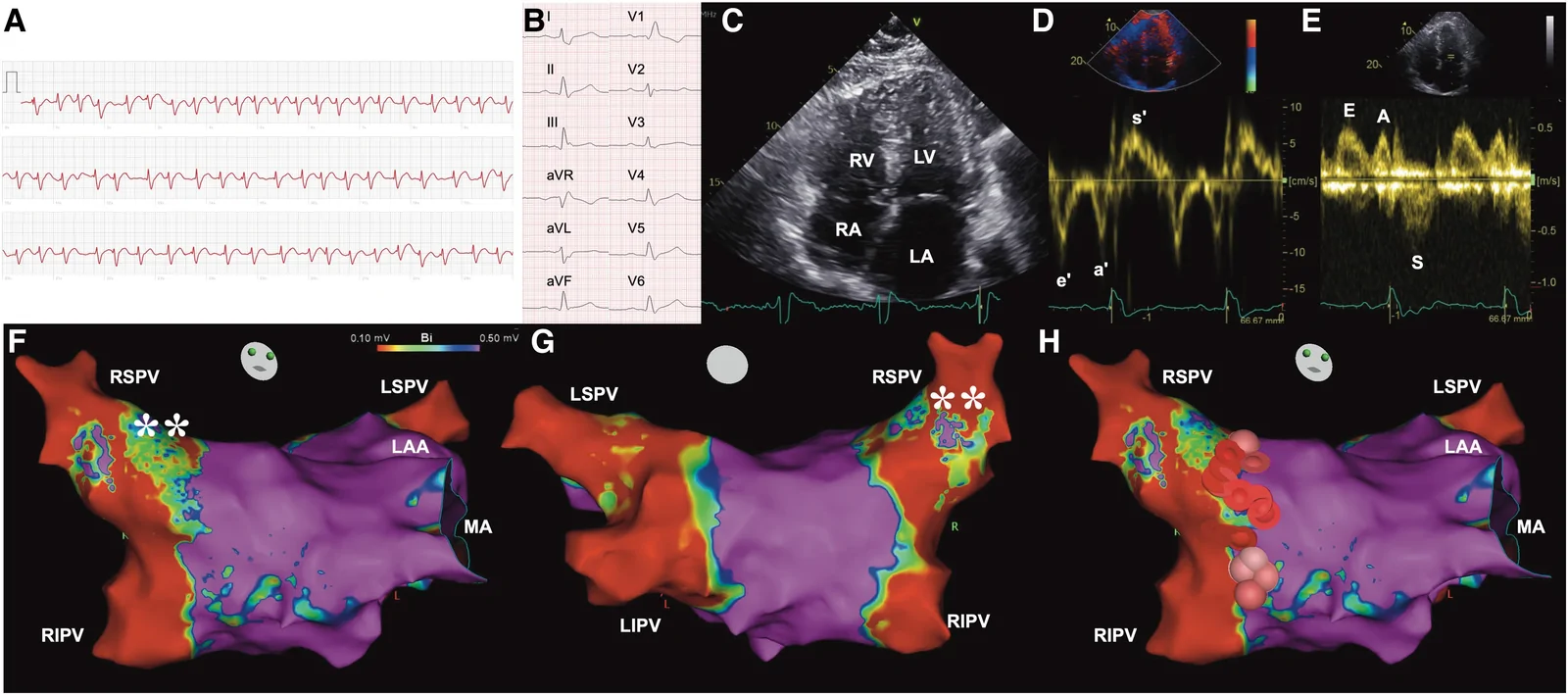
Clinical presentation, diagnostic findings, and procedural characteristics. (*A*) Smartwatch-derived single-lead echocardiography (ECG) documenting atrial fibrillation episodes during symptoms. (*B*) Resting 12-lead ECG in sinus rhythm. (*C–E*) Transthoracic echocardiography (*F*, *G*) Electroanatomical voltage maps from right anterior oblique view (*F*) and posterior view (*G*). (*H*) Ablation tags. LA, left atrium; LAA, left atrial appendage; LIPV, left inferior pulmonary vein; LSPV, left superior pulmonary vein; LV, left ventricle; MA, mitral anulus; RA, right atrium; RIPV, right inferior pulmonary vein; RSPV, right superior pulmonary vein; RV, right ventricle. **Reconnection site at the RSPV.

Despite procedural success, symptomatic AF episodes persisted (mEHRA class IIb–III). Flecainide had to be discontinued for intolerance. Rate control consisted of irregular bisoprolol intake, and no further antiarrhythmics or other medications were used. With a CHA_2_DS_2_-VA score of 1 (aHT), anticoagulation was limited to the peri-procedural periods surrounding pulmonary vein isolation and electrical cardioversion, as well as during documented AF episodes. The HAS-BLED score was 0, indicating no increased bleeding risk.

In January 2026, with body weight having increased to 117 kg despite repeated attempts at weight control through dietary modification and increased physical activity, semaglutide was initiated for obesity management at a dose of 0.25 mg/week and gradually escalated (see timeline). Episodes of AF and associated symptoms ceased following initiation of semaglutide treatment and have not recurred during follow-up (Summary Fig.). After 6 months, body weight had decreased by 14 kg. The patient reported mild gastrointestinal adverse effects including gastro-oesophageal reflux and nausea. Resting heart rate increased modestly from ∼70 to 80 b.p.m. Previously identified triggers of AF, such as moderate alcohol consumption, sleep deprivation, and large meals, no longer led to symptomatic arrhythmic episodes.

## Discussion

This case describes cessation of recurrent symptomatic AF following initiation of semaglutide in a patient with obesity and prior ablation failure. This temporal association is notable, particularly given the longstanding disease course and prior rhythm control interventions.

Obesity promotes atrial remodelling and inflammation, contributing to AF,^2,3^ and weight reduction improves rhythm control outcomes.^4^ The European Society of Cardiology guidelines emphasize weight reduction as a critical intervention to reduce AF burden.^1^ In the present case, a reduction in body weight by 14 kg was observed within 6 months of therapy initiation. However, the rapid cessation of AF episodes shortly after starting semaglutide suggests involvement of additional mechanisms.

Atrial fibrillation is increasingly recognized as a systemic disease closely linked to a continuum of metabolic dysfunction, obesity, chronic low grade systemic inflammation, and heart failure with preserved ejection fraction (HFpEF).^1^ In this context, cardiometabolic therapies may influence arrhythmia not only indirectly through risk factor modification but potentially also through direct myocardial effects. Experimental data suggest that GLP-1 receptors are widely expressed in atrial myocardium. In rabbit pulmonary vein cardiomyocytes, GLP-1 agonists reduce L-type Ca^2+^, NCX, and late Na^+^ currents, while increasing sarcoplasmic reticulum Ca^2+^ content.^8^ Furthermore, liraglutide reduced AF burden in a murine model of type 2 diabetes.^9^ In human ventricular cardiomyocytes, semaglutide has been shown to attenuate late Na^+^ currents and reduce diastolic Ca^2+^ sparks.^10^ Whether such direct electrophysiological effects contribute to rhythm stabilization in humans remains to be determined.

Moreover, rather than functioning as a conventional ion-channel modulating antiarrhythmic agent, semaglutide may act upstream by modulating metabolic, inflammatory, and profibrotic pathways or improving glycaemic control and autonomic balance.^8^

Large cardiovascular outcome trials have established cardioprotective effects of GLP-1 receptor agonists in patients with type 2 diabetes and in individuals with obesity and established cardiovascular disease.^6,7^ A large meta-analysis including 12 651 patients from 10 randomized controlled trials recently reported that semaglutide significantly reduced the incidence of AF by 42% in individuals at high cardiovascular risk.^11^ Finally, two recently published prospective studies demonstrated positive effects of semaglutide on AF recurrence in overweight patients following catheter ablation.^12,13^ In the STEP-HFpEF program, the magnitude of semaglutide-mediated improvements in heart failure-related symptoms and physical limitations were more pronounced in patients with AF than in those without. Unfortunately, no data were provided on AF conversion.^14^

Several alternative explanations should be considered, including cumulative effects of prior ablations, or β-blocker co-medication, temporal variability of AF, and unrecognized behavioural changes following initiation of weight management therapy. Further, no Holter monitoring was available, and the absence of recurrent AF was based on continuous smartwatch monitoring. This single-patient observation with limited follow-up is therefore not generalizable and requires confirmation with longer-term monitoring.

While causality cannot be established, the close temporal association between initiation of semaglutide therapy and cessation of recurrent AF in a patient with obesity and prior ablation failure suggests a potentially relevant interaction between cardiometabolic treatment and rhythm control. The observation may contribute to generating hypotheses and supports further prospective investigation into the role of GLP-1 receptor agonists in the management of HFpEF and obesity-associated AF.

## Lead author biography


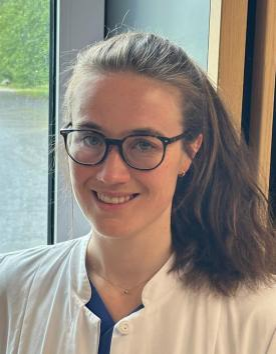


Dr. Frederike Marie Weitkamp studied medicine at Riga Stradins University and the Technical University Dresden, graduating in 2022. She continued her residency training in internal medicine at the Spitalzentrum Oberwallis, Switzerland and the Clemenshospital, Münster and since 2025 is a cardiology and cardiac electrophysiology fellow at the University Medical Center Göttingen, Germany. Her scientific interests focus on atrial fibrillation, cardiometabolic disease, and translational electrophysiology.

## Supplementary Material

ytag727_Supplementary_Data

## Data Availability

All available data are presented within the manuscript.

## Notes

**Consent:** Written informed consent was obtained from the patient for publication of this case report and any accompanying images in accordance with journal requirements.
