# Supplementary material for: Cardiometabolic therapy for rhythm control: a case report of refractory atrial fibrillation resolving after initiation of semaglutide for obesity
Source: Eur Heart J Case Rep. 2026 Sep 28;10(10):ytag727. doi: 10.1093/ehjcr/ytag727 (PMC13662400; doi:10.1093/ehjcr/ytag727)
Supplement: Supplementary file 1 [file ytag727_supplementary_data.zip › Online supplement 02.8.2026_fw.docx]

|  | Parameter | Result | Reference |
| --- | --- | --- | --- |
| **Left ventricle** | LV end-diastolic diameter | 52 mm | ≤55 mm |
|  | LV end-systolic diameter | 37 mm | ≤35 mm |
|  | Interventricular septal thickness | 12 mm | <11 mm |
|  | Posterior wall thickness | 9 mm | <10 mm |
|  | LV ejection fraction | 60% |  |
|  | Mitral annular plane systolic excursion | 1.5 cm | ≥10 mm |
|  | Regional wall motion | No abnormalities |  |
|  | LV geometry | Normal-sized LV with mild septal-predominant hypertrophy |  |
| **LV diastolic function** | Mitral E/A ratio | 1.2 |  |
|  | E-wave deceleration time | 230 ms | 160–240 ms |
|  | Average E/e′ ratio | 5 | <8 |
| **Left atrium** | Anteroposterior diameter | 45 mm | <40 mm |
| **Right ventricle** | RV diameter | 36 mm | ≤39 mm |
|  | TAPSE | 1.9 cm | ≥17 mm |
| Right atrium | Right atrial size | Qualitatively normal |  |
| **Pulmonary pressure and vena cava** | Estimated systolic pulmonary artery pressure | 22 mmHg | ≤35 mmHg |
|  | Estimated right atrial pressure | 5 mmHg | ≤5 mmHg |
|  | Inferior vena cava diameter | 16 mm | <20 mm |
|  | Respiratory IVC collapse | ≥50% |  |
| **Aorta** | Aortic root diameter | 32 mm | Normal, ≤38 mm |
|  | Ascending aorta | Qualitatively normal where assessable |  |
| **Valves** | Aortic valve | Tricuspid, mildly thickened, with preserved opening |  |
|  | Mitral valve | Mildly thickened, with preserved opening |  |
|  | Tricuspid valve | — |  |
|  | Other valves | Sonographically and Doppler-normal |  |
| **Pericardium** | Pericardial effusion | None |  |

***Supplement Table S1: Echocardiographic findings***

IVC, inferior vena cava; LA, left atrial; LV, left ventricular; RV, right ventricular; TAPSE, tricuspid annular plane systolic excursion.
